# Index tumor location affected early biochemical recurrence after radical prostatectomy in patients with negative surgical margin: a retrospective study

**DOI:** 10.1186/s12894-024-01499-4

**Published:** 2024-05-18

**Authors:** Yoshihiko Ogata, Jun Akatsuka, Yuki Endo, Hikaru Mikami, Masato Yanagi, Hayato Takeda, Yuka Toyama, Yoichiro Yamamoto, Go Kimura, Yukihiro Kondo

**Affiliations:** 1 Department of Urology, Nippon Medical School Tama Nagayama Hospital, Tokyo, 206-8512 Japan; 2 Department of Urology, Nippon Medical School Hospital, 1-1-5 Sendagi, Bunkyo-ku, Tokyo, 113-8603 Japan; 3 Pathology Informatics Team, RIKEN Center for Advanced Intelligence Project, Tokyo, 103-0027 Japan; 4 Mathematical Intelligence for Medicine, Graduate School of Medicine, Tohoku University, Miyagi, 980-8574 Japan

**Keywords:** Prostate neoplasms, Index tumor, Surgical pathology, Prostatectomy

## Abstract

**Background:**

Index tumors are the most aggressive tumors of the prostate. However, their clinical significance remains unclear. This study aimed to assess the incidence of index tumor location according to the zonal origin and whether these locations affect the prognosis after radical prostatectomy in patients with negative surgical margins.

**Methods:**

This single-centered, retrospective study evaluated 1,109 consecutive patients who underwent radical prostatectomies. An index tumor was defined as the largest tumor in the prostate gland. We detected these locations based on McNeal's zonal origin using whole-mount sections. Biochemical recurrence (BCR) free survival curves were generated using the Kaplan–Meier method. Univariate and multivariate analyses using the Cox proportional hazards model were performed to determine the predictive factors for early BCR (within 1-year).

**Results:**

A total of 621 patients with negative surgical margins who did not receive adjuvant therapy were included in this study. The index tumor were located in the transitional zone in 191 patients (30.8%), the peripheral zone in 399 patients (64.3%), and the central zone in 31 patients (5.0%). In total, 22 of 621 patients (3.5%) experienced early BCR and 70 patients (11.2%) experienced overall BCR at a median follow-up of 61.7 months. According to the index tumor location, the early BCR-free rates were 99.5%, 95.7 %, and 83.3% in the transitional, peripheral, and central zones, respectively. On multivariate analysis, the index tumor in the central zone was an independent predictor of early BCR with negative surgical margins following radical prostatectomy, followed by prostatectomy pathological grade, index tumor in the peripheral zone, and high prostate-specific antigen level.

**Conclusions:**

We assessed the significance of index tumor location in patients with negative surgical margins following radical prostatectomy. Index tumors located in the central zone, although infrequent, were the strongest predictive factors for early BCR. Our results may allow urologists and patients to reconsider the therapeutic strategies for prostate cancer.

**Supplementary Information:**

The online version contains supplementary material available at 10.1186/s12894-024-01499-4.

## Background

Radical prostatectomy is the standard treatment for localized prostate cancer. The primary goal of radical prostatectomy is to achieve negative surgical margins, indicating the absence of cancer cells. However, some patients experience biochemical recurrence (BCR) after radical prostatectomy, even with negative surgical margins [1–5]. Robotic surgery has recently become mainstream in radical prostatectomy; therefore, the number of patients with negative surgical margins will continue to increase. It is crucial to identify the predictive factors of BCR in these patients for individual cancer management.

Prostate cancer has a multifocal nodule in most patients within the prostate [6–8]. Each nodule within the prostate gland exhibited an independent pathological pattern, supporting a clonal origin at the molecular and genetic levels. Index tumors were first introduced by McNeal *et al*, and refer to tumor nodules that are most likely to demonstrate more aggressive biological behavior among the multifocal tumor nodules within the prostate [9]. However, their clinical significance remains unclear.

Cancer location affects the management in various oncology fields. However, only a few reports have discussed the locational significance for prostate cancer, not leading to the conclusive evidence [10–12]. Evaluating the significance of index tumor location in radical prostatectomy with surgical complete resection may influence treatment decisions for localized prostate cancer. Therefore, this study aimed to assess the incidence of index tumor location according to the zonal origin and whether these locations affect the prognosis after radical prostatectomy in patients with negative surgical margins.

## Methods

### Patient population

This single-centered, retrospective study obtained data of patients with localized prostate cancer who underwent radical prostatectomy at our institution (Nippon Medical School Hospital: NMSH) between 2000 and 2018. A cohort of 1,109 patients was identified. Then, we excluded patients who received adjuvant therapy, including androgen deprivation and/or radiotherapy. Finally, we evaluated patients after radical prostatectomy with a negative surgical margin and sufficient clinical course. This study was approved by the Institutional Review Boards of the NMSH (reference 28-11-663) and was conducted in accordance with the tenets of the Declaration of Helsinki. The need for written informed consent was waived by the Ethics Committee at NMSH, based on the retrospective nature of the study. However, all participants had the opportunity to opt-out on a homepage of the Ethics Committee at NMSH.

### Pathological examination

For all patients, we prepared serial whole-mounted sections with a 3-5-mm thickness of the prostate gland, resected in a direction perpendicular to the rectal surface from the prostate apex to the bladder neck. All samples were sectioned at 3-μm thickness and stained with hematoxylin and eosin. An expert genitourinary pathologist reviewed all surgical pathology slides in a blinded fashion according to the 2014 International Society of Urological Pathology (ISUP) grading system [13]. The pathologist marked the cancer foci on the pathological slides from each serial whole-mount section (supplemental Figure 1). Currently, index tumors are the largest tumor nodules among multifocal nodules [7, 8]. Therefore, we defined the largest nodule as the index tumor with reference to the major and minor axes of the cancer foci on cancer mapping by using serial whole-mount section.

### Follow up and outcomes

Serum prostate-specific antigen (PSA) levels were measured once within the first 6 weeks postoperatively and every 3 months thereafter. In this study, BCR was defined as having a PSA level > 0.2 ng/mL postoperatively, confirmed in at least two consecutive tests. If the postoperative PSA values were not < 0.2 ng/mL at routine postoperative follow-up, the surgery date was noted as the recurrence date. Our concern was early BCR after radical prostatectomy because it is associated with poor clinical prognosis [14]. Therefore, we defined BCR after radical prostatectomy in the first year as early BCR for our analysis.

### Statistical analysis

We summarized the data on patient demographics, tumor characteristics, and index tumor location in the prostate gland using descriptive statistics. We used the Wilcoxon test for continuous variables and Fisher’s exact test for categorical data. Descriptive statistics were calculated as the average and standard deviation (age, body mass index, and total prostate volume), and median and interquartile range (follow-up period). BCR-free survival was determined using the Kaplan–Meier method. Survival curves were stratified by the index tumor location and compared using a two-sided log-rank test. Factors associated with early BCR (within the first year) and overall BCR (during the follow-up period) were evaluated using Cox proportional hazards models. All statistical analyses were done using JMPv13.0 software (SAS Institute Inc., Cary, NC, USA). All analyses with 2-sided *p* < 0.05, which was considered statistically significant.

## Results

**Fig. 1:**
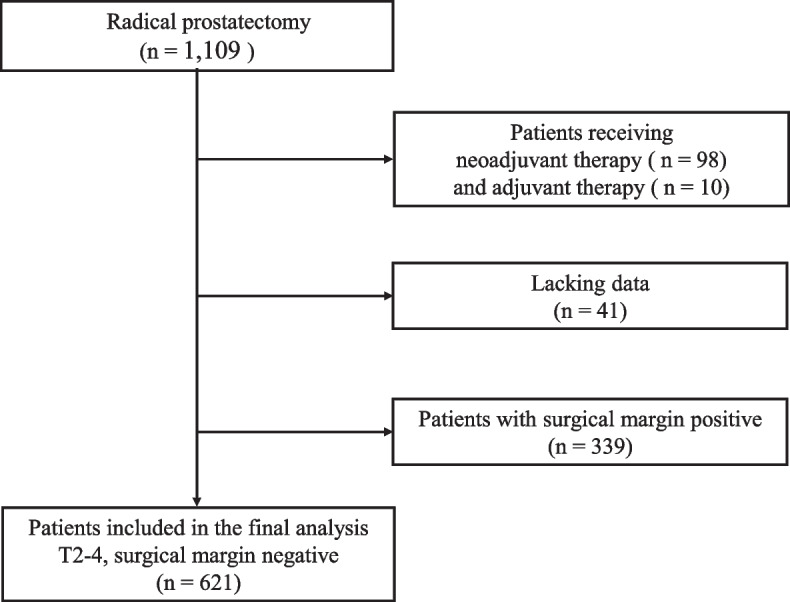
Flow diagram of the study

The study profiles are shown in Fig. 1. We excluded patients with a history of neoadjuvant therapy (98 patients), adjuvant therapy (10 patients), or insufficient data (41 patients including in whom the location of the index tumor was not identified). Finally, 621 patients with negative surgical margins after radical prostatectomy for NMSH were evaluated, all of whom were of Asian descent. Table 1 shows the clinical and pathological characteristics and Table 2 shows a comparative analysis of the index tumor locations. We identified the location of the index tumor in transitional zone 191 (30.8%), peripheral zone 399 (64.3%), or central zone 31 (5.0%). Regarding the preoperative factors, the average age was 66.5 ± 6.2 years, and the average BMI was 23.5 ± 2.8 kg/m^2^, which were not significantly different among the three index tumor locations. PSA level was ≤ 10 in 430 out of 621 patients (69.2%), 10–20 in 152 patients (24.5%), and > 20 in 39 patients (6.3%). Index tumors in the transitional zone had significantly higher PSA levels than those in the peripheral zone (*P* = 0.0003). In contrast, the clinical T stage in the transitional zone was significantly lower than that in other index tumor locations (vs. the peripheral zone, *p* = 0.004, vs central zone, *p* = 0.001). Regarding postoperative factors, index tumors located in the central zone were of significantly higher prostatectomy ISUP grades and pathological T stages (both *p* < 0.05) when compared to those in other index tumor locations, as evidenced by pathological extraprostatic extension, seminal vesicle invasion, and bladder neck involvement. The significance of lymph node dissection in radical prostatectomy varies [15]. At our institution, the attending physician performed lymph node dissection based on the patient's cancer status and treatment era. Similarl to the other post-operative factors, patients with index tumors in the central zone demonstrated significantly higher rates of pathological lymph node metastasis than those with other index tumor locations (*p* < 0.05).

**Table 1:** Characteristics of enrolled patients: entire cohort

Patient in the entire cohort: *N* = 621			
Age, y/o average ±SD		66.5	±6.2
BMI, kg/m2 average ± SD		23.5	±2.8
TPV, ml average ± SD		30.4	±13.5
Initial PSA (ng/ml) n, (%)	≤10	430	69.2
	10-20	152	24.5
	>20	39	6.3
cT stage n, (%)	T1	173	27.9
	T2	406	65.4
	T3-4	42	6.8
Biopsy ISUP grade n, (%)	1-2	349	56.2
	3	115	18.5
	4	96	15.5
	5	61	9.8
Prostatectomy ISUP grade n, (%)	1-2	303	48.8
	3	127	20.5
	4	94	15.1
	5	97	15.6
pT stage n, (%)	T2	485	78.1
	T3	123	19.8
	T4	13	2.1
pEPE		126	20.3
pSVI		18	2.9
pN n, (%)		2	0.3

*Abbreviations*: *SD* standard deviation, *BMI* body mass index, *PSA* prostate-specific antigen, *cT* clinical T stage, *pT* pathological T stage, *ISUP* International Society of Urological Pathology, *TPV* total prostate volume, *pEPE* pathological extraprostatic extension, *pSVI* pathological seminal vesicle invasion, *pN* pathological lymph node metastasis

**Table 2:** Characteristics of patients according to index tumor location

		Transitional zone (191:30.8%)		Peripheral zone (399:64.3%)		Central zone (31:5.0%)		Transitional zone vs. Peripheral zone	Transitional zone vs. Central zone	Peripheral zone vs. Central zone
Age (years) average ±SD		66.5	±5.9	66.6	±6.3	65.9	±6.7	0.48	0.77	0.57
BMI, kg/m2 average ± SD		23.6	±2.9	23.5	±2.7	23.7	±2.5	0.85	0.47	0.45
Initial PSA (ng/ml) n (%)	≤10	116	60.7	296	74.2	18	58.1	0.0003	0.23	0.10
	10-20	53	27.8	87	21.8	12	38.7			
	>20	22	11.5	16	4.0	1	3.2			
cT stage n (%)	T1	65	34.0	106	26.6	2	6.5	0.004	0.001	0.04
	T2	122	63.9	258	64.7	26	83.9			
	T3-4	4	2.1	35	8.8	3	9.7			
Biopsy ISUP grade n (%)	1-2	115	60.2	223	55.9	11	35.5	0.56	0.003	0.09
	3	36	18.9	73	18.3	6	19.4			
	4	23	12.0	65	16.3	8	25.8			
	5	17	8.9	38	9.5	6	19.4			
Prostatectomy ISUP grade n (%)	1-2	108	56.5	188	47.1	7	22.6	0.11	0.004	0.02
	3	34	17.8	84	21.1	9	29.0			
	4	21	11.0	68	17.0	5	16.1			
	5	28	14.7	59	14.8	10	32.3			
pT stage n (%)	T2	160	83.8	309	77.4	16	51.6	0.001	< 0.0001	0.005
	T3	23	12.0	86	21.6	14	45.2			
	T4	8	4.2	4	1.0	1	3.2			
pEPE		25	13.1	88	22.1	13	41.9	0.01	0.0003	0.03
pSVI		2	1.1	10	2.5	6	19.4	0.35	0.0001	0.0004
pN n (%)		0	0.0	2	0.5	2	6.5	-	0.0004	< 0.0001

*Abbreviations*: *SD* standard deviation, *BMI* body mass index, *PSA* prostate-specific antigen, *cT* clinical T stage, *pT* pathological T stage, *ISUP* International Society of Urological Pathology, *pEPE* pathological extraprostatic extension, *pSVI* pathological seminal vesicle invasion, *pN* pathological lymph node metastasis

**Fig. 2:**
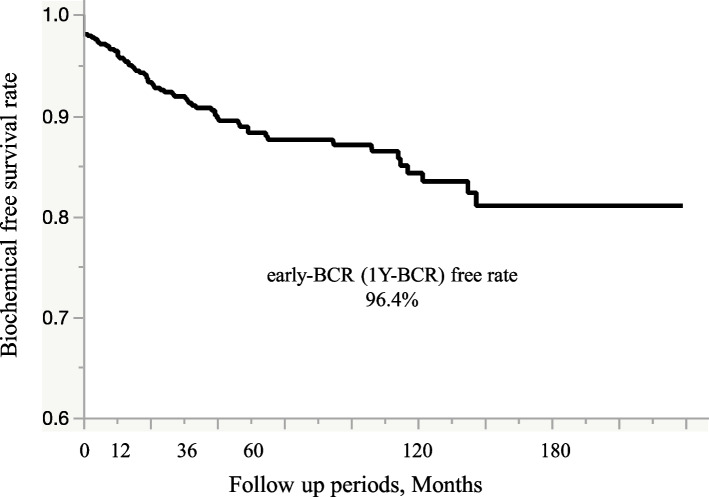
Kaplan–Meier Curve in entire cohort. Kaplan**–**Meier curve of biochemical recurrence-free survival rate. (One-year biochemical recurrence-free probability,96.4%; 5-yr recurrence-free probability,88.3%; 10-yr recurrence-free probability,84.3%)

**Fig. 3:**
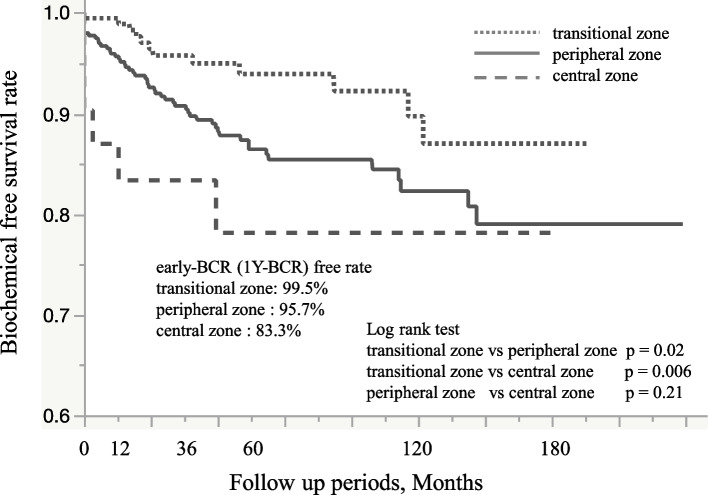
Kaplan–Meier Curve according to index tumor location. The dotted line indicates the Kaplan-Meier curve index of the tumor in the transitional zone. The gray line indicates the Kaplan-Meier curve index of the tumor in the peripheral zone. The long dashed line indicates the Kaplan-Meier curve index tumor in the central zone. (One-year biochemical recurrence-free probability:99.5%, 95.7 %, and 83.3% in the transitional, peripheral, and central zones, respectively). Log-rank test: transitional zone vs. peripheral zone, *p* = 0.02; transitional zone vs. central zone, *p* = 0.006; peripheral zone vs. central zone, *p* = 0.21)

Median follow-up was 61.7 months [interquartile range 33.3–105.5 months]. During the follow-up period, 22 of the 621 patients (3.5%) experienced early BCR and 70 (11.2%) experienced all-BCR. Figure 2 shows the Kaplan–Meier curve showing BCR-free survival. and the 1-, 5-, and 10-year BCR-free survival rates were 96.4%, 88.3%, and 84.3%, respectively, in all groups. Figure 3 shows the Kaplan–Meier curve according to the index tumor locations. One-year BCR free survival rates in the transitional, peripheral, and central zones were 99.5 %, 95.7%, and 83.3%, respectively. Index tumor in the transitional zone was significantly better prognosis among three locations (log-rank test vs. peripheral zone: *p* = 0.002; vs. central zone: *p* = 0.006). In the univariate analysis, initial PSA level, prostatectomy ISUP grade, pathological T stage, lymph node metastasis, and index tumor location were significant predictive factors for early BCR. In the multivariate analysis, we identified three independent predictors of early BCR (PSA level, prostatectomy ISUP grade, and index tumor location) after radical prostatectomy (Table 3). An index tumor in the central zone was the strongest prognostic factor of early BCR (Hazard ratio 16, *p* = 0.02), followed by prostatectomy ISUP grade, index tumor in the peripheral zone, and high PSA level. Supplemental Table 1 shows the analysis of overall BCR. These analyses identified preoperative PSA level, lymph node metastasis, pathological T stage, age, and prostatectomy ISUP grade as the major contributors to this poor prognosis, rather than the index tumor location. Following BCR after radical prostatectomy, the attending physician administered salvage radiation therapy and hormone therapy. Consequently, there were no instances of mortality attributable to prostate cancer among these patients during the observation period.

**Table 3:** Univariate and multivariate analysis of clinicopathological factors for early-BCR FS for radical prostatectomy patients

Variable		Univariate HR (95% CI)	*p* value	Multivariate HR (95% CI)	*p* value
Age, y/o	(67 > vs 67 ≤)	0.8 (0.4-1.9)	0.8		
BMI, kg/m2	(23.6 > vs 23.6 ≤)	0.9 (0.4-2.2)	0.9		
Initial PSA (ng/ml)	≤10	ref	-	-	-
	10-20	4.5 (1.8-12)	0.001	4.2 (1.6-11)	0.003
	>20	6.4 (1.9-22)	0.003	7.4 (2.1-26)	0.002
Prostatectomy ISUP grade	1-2	Ref	-	-	-
	3	4.9 (0.9-27)	0.07	-	-
	4	6.5 (1.2-36)	0.03	-	-
	5	20.0(4.4-88)	< 0.0001	13.1 (2.9-60)	0.0009
pT stage	T2	Ref	-		
	T3-4	4.4 (1.9-10.2)	0.0005		
pN	(+ vs -)	18.8 (2.5-140)	0.004		
Index tumor location	transitional zone	-	-	-	-
	peripheral zone	8.1(1.1-12)	0.04	12 (1.5-93)	0.02
	central zone	26.0 (2.9-233)	0.004	16 (1.6-167)	0.02

*Abbreviations*: *BCR-FS* biochemical recurrence-free survival, *BMI* body mass index, *PSA* prostate-specific antigen, *pT* pathological T stage, *ISUP* International Society of Urological Pathology, *pN* pathological lymph node metastasis, *HR* Hazard ratio, *CI* confidence interval

## Discussion

We evaluated the clinical significance of index tumor location after radical prostatectomy with a negative surgical margin using whole-mount section analysis. Index tumors located in the central zone, although infrequent, were the strongest prognostic factors for early BCR, even if the surgical margin was negative.

A negative surgical margin is imperative for urological surgeons because it enables a cancer-free status. However, some patients experience BCR even with negative surgical margins, at a rate is about 87-96% [5, 16, 17]. The preoperative serum PSA level, Gleason score, pathological T stage, and margin status are widely accepted as significant prognostic factors in those patients [3–5]. A report from the USA showed significant risk factors for BCR in patients with organ-confined prostate cancer and negative surgical margins, including African American race, suspicious digital rectal examination, serum PSA level, and ISUP grades 4 and 5 on prostatectomy pathology [16]. In a retrospective multicenter cohort study conducted in Belgium, the 5- and 10-year BCR-free survival rates were 90% and 87%, respectively [5]. On the multivariate analyses, serum PSA level and pathological Gleason score of 7 or above were significantly associated with BCR in men with organ-confined prostate cancer with negative surgical margins. Oikawa et al. reported that the 5- and 10-year BCR-free survival rates were 89.0% and 87.8% after radical prostatectomy in patients with those statuses [3]. Multivariate analysis revealed that the proportion of biopsy-positive cores was significantly associated with BCR. In contrast, biopsy Gleason score and D’Amico risk classification were not significant predictive factors. Our study showed that the 1-, 5-, and 10-year BCR-free survival rates were 96.4%, 88.3%, and 84.3%, respectively. We evaluated the predictive factors of early BCR (within 1-year), adding the index tumor location to the general clinicopathological factors. Consequently, an index tumor in the central zone was the strongest prognostic factor for early BCR after radical prostatectomy in patients with negative surgical margins, which did not lead to overall BCR.

Index tumors were first introduced by McNeal et al., and refer to tumor nodules that are most likely to exhibit more aggressive biological behavior among the multifocal tumor nodules within the prostate [9]. A previous study revealed a correlation between the index tumor location and pathological features within the prostate. Billis et al. showed that index tumors with a predominantly posterior location were significantly associated with a higher total tumor extent, biopsy and prostatectomy Gleason score, lymph node metastasis, and preoperative PSA level [18]. Van de Voorde et al. found that extraprostatic extension, seminal vesicle involvement, surgical margin status, and lymph node metastasis were observed in 33 %, 17 %, 29 %, and 4% of index tumors in the transitional zone, respectively, compared with 58%, 20%, 48%, and 6% of index tumors in the peripheral zone [19]. In addition, O’Neil et al. compared transitional zone tumors with peripheral zone tumors and found that the former was larger, more frequently lower grade, organ-confined, and preferentially involved the bladder neck (49% vs. 6%, *p* < 0.001) [20]. Cohen et al. performed a characterization and comparative analysis of central zone carcinomas [12]. They detected 1,767 index tumors of zonal origin: 1,360 (77%) in the peripheral zone, 348 (19.7%) in the transitional zone, and 59 (3.3%) in the central zone. Cancer in the central zone is significantly more aggressive than that in other zonal locations, with a high risk of worse pathological factors and margin status. Our study showed that index tumors located in the central zone, although infrequent (5.0%), had significantly worse clinical and pathological features among the three locations within the prostate gland in patients with negative surgical margins. In recent years, there has been significant progress in developing nomograms for evaluating the presence of index tumors [21], as well as in studies aimed at extracting the location information of these tumors [22]. Future comprehensive studies on index tumors in prostate cancer will strengthen our results.

The central zone is located around the ejaculatory duct, contacts the bladder on the ventral side, and penetrates the nerve on the posterolateral side [23]. Therefore, prostate cancer that is localized in the central zone easily progresses outside the prostate gland and exhibits lymphovascular invasion. Prostate cancer localized in the central zone is relatively rare; however, it tends to be more aggressive than that in other zones [12]. Our study demonstrates the significance of the index tumor location and suggests the following therapeutic strategies. Adjuvant therapy may be required if pathology after prostatectomy reveals an index tumor in the central zone, even with complete surgical resection. Furthermore, if an index tumor in the central zone is suggested during preoperative evaluation, a multimodal treatment combining neoadjuvant hormonal therapy and/or chemotherapy with radical prostatectomy may be required. Recently, index tumors have been evaluated preoperatively using radiological and biopsy approach [24, 25]. Our results may allow urologists and patients to reconsider the therapeutic strategies for prostate cancer.

This study had several limitations. First, our study was a retrospective investigation conducted at a single medical facility. Considering the complexity introduced by this study design, generalizing our results to a broader population may be challenging. Furthermore, potential biases in patient selection could impact our outcomes, potentially affecting the overall reliability of our results. Second, in our cohort, the attending physician determined the necessity and extent of lymph node dissection during radical prostatectomy based on the patient’s cancer status and treatment era, revealing that only 0.3% of patients had lymph node metastasis. Indications for lymph node dissection vary by institution, and our data reflect real-world settings in a single Japanese medical center. Third, in our analysis, all patients were of Asian descent. It is recognized that biological characteristics and responsiveness to treatment in prostate cancer may vary among different racial groups [26]. Therefore, it is imperative to acknowledge the potential influence of racial differences when interpreting our results. While several limitations exist, our results are highly uniform and reliable for surveys, because we collected our data from a single team conducting radical prostatectomies, and a single pathologist diagnosed all the whole-mount sections. In the future, large-scale multicenter analyses and prospective studies are required to resolve these limitations and establish a more robust analysis.

## Conclusions

Our study provides information on the incidence and prognosis of index tumor locations after radical prostatectomy with complete surgical resection. The index tumor in the central zone had the lowest frequency, whereas its location had the highest risk of recurrence for early BCR. Index tumor location information may influence physicians in attaining adequate cancer management for localized prostate cancer.

### Supplementary Information

Supplementary Material 1.

Supplementary Material 2.

## Data Availability

The datasets used and/or analysed during the current study are available from the corresponding author on reasonable request.

## Abbreviations

NMSH: Nippon Medical School Hospital
PSA: Prostate specific antigen
BCR: Biochemical recurrence
ISUP: International Society of Urological Pathology
