# Supplementary material for: Index tumor location affected early biochemical recurrence after radical prostatectomy in patients with negative surgical margin: a retrospective study
Source: BMC Urol. 2024 May 18;24:108. doi: 10.1186/s12894-024-01499-4 (PMC11102263; doi:10.1186/s12894-024-01499-4)
Supplement: Supplementary file 1 — Supplementary Material 1. [file 12894_2024_1499_MOESM1_ESM.pdf]

Supplemental Figure 1.

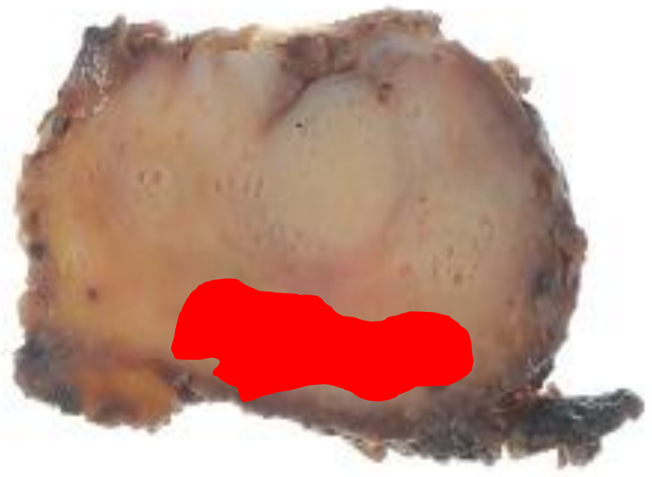

Supplemental Figure 1. Whole mount map of radical prostatectomy specimen

Whole mount section from the base of the prostate. The index tumor was located in the red-marked area, specifically within the central zone.
