# Supplementary material for: Index tumor location affected early biochemical recurrence after radical prostatectomy in patients with negative surgical margin: a retrospective study
Source: BMC Urol. 2024 May 18;24:108. doi: 10.1186/s12894-024-01499-4 (PMC11102263; doi:10.1186/s12894-024-01499-4)
Supplement: Supplementary file 2 — Supplementary Material 2. [file 12894_2024_1499_MOESM2_ESM.docx]

**Additional File**

**Supplemental Table1 Univariate and Multivariate analysis of clinicopathological factors for over all-BCR FS for radical prostatectomy patients**

| Variable | | Univariate HR  (95% CI) | p value | Multivariate HR  (95% CI) | p value |
| --- | --- | --- | --- | --- | --- |
| Age, y/o | (67 > vs 67 ≤) | 0.5 (0.3-0.9) | 0.01 | 0.5(0.3-0.8) | 0.003 |
| BMI, kg/m2 | (23.6 > vs 23.6 ≤) | 1.4 (0.8-2.2) | 0.2 |  |  |
| Initial PSA (ng/ml) | ≤ 10  10-20   - 20 | ref  4.5 (1.8-12)  6.4 (1.9-22) | -  0.002  0.003 | -  2.2 (1.3-3.8)  - | -  0.004  - |
| Prostatectomy  ISUP grade | 1-2  3  4  5 | Ref  2.7 (1.2-6.1)  5.5 (2.6-12)  10.2 (5.2-20) | -  0.01  < 0.0001  < 0.0001 | -  -  4.0 (1.8-8.5)  8.2 (4.0-17) | -  -  0.0004  < 0.0001 |
| pT stage | T2  T3-4 | Ref  4.2 (2.6-6.7) | -  < 0.0001 | -  2.3 (1.4-3.9) | -  0.002 |
| pN | (+ vs -) | 7.1 (1.0-51) | 0.053 | 8.4 (1.2-61) | 0.04 |
| Index tumor location | transitional zone  peripheral zone  central zone | -  2.0 (1.1-3.8）  3.5 (1.3-9.4) | -  0.03  0.01 | -  2.2 (1.2-4.2)  - | -  0.02  - |

Abbreviations: BCR-FS, biochemical recurrence-free survival; BMI, body mass index; PSA, prostate-specific antigen; pT, pathological T stage; ISUP, International Society of Urological Pathology; pN, pathological lymph node metastasis; HR: Hazard ratio; CI, confidence interval
